# Increase in Extraintestinal Infections Caused by *Salmonella enterica* Subspecies II–IV

**DOI:** 10.3201/eid1804.111386

**Published:** 2012-04

**Authors:** Sharon L. Abbott, Frank C.Y. Ni, J. Michael Janda

**Affiliations:** California Department of Public Health, Richmond, California, USA (S.L. Abbott, F.C.Y. Ni, J.M. Janda);; Los Angeles County Public Health Laboratory, Downey, California, USA (J.M. Janda).

**Keywords:** Salmonellae, Salmonella enterica, subspecies II–IV, extraintestinal infections, epidemiology, demographics, bacteria, enteric infections, California, United States

## Abstract

To garner information regarding site of infection and age and sex of persons infected with *Salmonella enterica* subspecies II–IV, we retrospectively analyzed data on *Salmonella* spp. infections in California, USA, 1985–2009. These subspecies were found to cause significantly more frequent invasive disease (e.g., bacteremia) than did *Salmonella* subspecies I strains.

The genus *Salmonella* has 2 species, *bongori* and *enterica*; the latter species is divided into 6 subspecies: *enterica* (I), *salamae* (II), *arizonae* (IIIa), *diarizonae* (IIIb), *houtenae* (IV), and *indica* (VI). *S*. *bongori* and *Salmonella* subsp. VI are rarely isolated from humans (*1*). However, for the more commonly isolated subspecies (II, IIIa, IIIb, IV), little information is available on their distribution in extraintestinal infections or on the demographic characteristics of the patients from whom they are recovered. Therefore, we retrospectively analyzed salmonellae data (>75,000 isolates) collected by a large state laboratory over 25 years.

## The Study

From 1985 through 2009, the Microbial Diseases Laboratory of the California Department of Public Health serotyped 1,342 isolates of *Salmonella* from individual patients; the isolates belonged to subspecies II–IV and encompassed 126 different serotypes. Serotypes of subspecies II were the most rare, with a total of 60 (4%) isolates; subspecies IIIa, IIIb, and IV had isolate totals of 463 (35%), 443 (33%), and 376 (28%), respectively. Overall, patients were more likely to have had subspecies II–IV isolated from feces (n = 947) than from an extraintestinal site (n = 395; p<0.001). Only patients from whom subspecies IIIa was isolated were equally likely to have had an extraintestinal infection as to have had diarrhea only (Table 1). Sources of extraintestinal infections included cerebrospinal fluid, blood, urine, cervix, bile, wounds and abscesses, and the respiratory tract. Blood and urine were the most common extraintestinal sites, comprising 42% (n = 167) and 33% (n = 129) of the isolates. Although blood was the most common site for subspecies IIIa and IV (p<0.001), the most common site for subspecies II and IIIb was urine (p<0.001). The only 2 subspecies isolated from cerebrospinal fluid were subspecies IIIa serotypes.

**Table 1:** Location of infections caused by salmonellae other than subspecies I among 1,342 patients, California, USA, 1985–2009

Site	% Subspecies				% Total, n = 1,342
	II, n = 60	IIIa, n = 463	IIIb, n = 443	IV, n = 376	
Feces*	85	33	59	79	71
Extraintestinal*	15	67	41	21	29
Blood†	0	51	19	57	42
Urine†	80	21	60	18	33
Wound/abscess†	0	7	4	5	6
Respiratory tract†	0	5	10	2	6
Other‡	20	15	6	18	13
Cerebrospinal fluid	0	5	0	0	0.5

*Percentage of total of each subspecies relative to intestinal or extraintestinal sites. †Percentage of total within each subspecies. ‡Includes genital tract, eye, tissue, gallbladder, and ascites fluid.

When information on the patient’s sex was included, male patients were only slightly more likely (707/1,342, 53%) than were female patients (617/1,342, 46%) to have had an infection caused by non–subspecies I salmonellae. The numbers of male and female patients were also comparable, regardless of whether the site of salmonellae infection was fecal or extraintestinal (Table 2). Only when patient’s sex was considered according to individual subspecies did the number of infections associated with male patients differ significantly from the number associated with female patients. Male patients were more likely than female patients to have fecal (63% vs. 36%; p>0.001) and extraintestinal (66% vs. 33%; p<0.001) infections caused by subspecies IIIa, whereas female patients were more likely to have extraintestinal infections caused by subspecies IIIb (68% vs. 31%; p<0.001).

**Table 2:** Distribution of infections caused by salmonellae other than subspecies I, by sex and age, among 1,342 patients, California, USA, 1985–2009

Subspecies	No. (%) patients by sex			No. (%) patients by age group, y			
	M	F		<1	2–10	11–60	>61
II							
Feces	25 (45)	29 (53)		31 (56)	11 (20)	7 (13)	0
Extraintestinal	1 (20)	4 (80)		1 (20)	0	4 (80)	0
IIIa							
Feces	146 (63)	84 (36)		30 (13)	108 (47)	32 (14)	8 (3)
Extraintestinal	154 (66)	77 (33)		4 (2)	126 (54)	79 (34)	15 (6)
IIIb							
Feces	160 (48)	161 (49)		48 (15)	155 (47)	44 (13)	18 (5)
Extraintestinal	35 (31)	77 (68)		9 (8)	56 (50)	36 (32)	6 (5)
IV							
Feces	168 (50)	159 (48)		173 (52)	46 (14)	67 (20)	15 (5)
Extraintestinal	18 (41)	26 (59)		12 (27)	9 (21)	15 (34)	7 (16)
Total*	707 (53)	617 (46)		349 (26)	157 (12)	538 (40)	213 (16)
Feces	499 (53)	433 (46)		321 (34)	135 (14)	337 (35)	91 (10)
Extraintestinal	208 (53)	184 (46)		28 (7)	22 (6)	201 (51)	122 (31)

*Numbers do not total 1,342 because data were not available for some patients.

Few remarkable associations were observed between patient age, site of infection, and specific subspecies (Table 2). In general, patients in the 11- to 60-year-old age group were more likely to have disease caused by subspecies IIIa or IIIb, whereas those <1 year of age were more likely to have infections caused by subspecies II or IV. Notably, however, 82% of all extraintestinal infections occurred in persons 11–60 (51%) or >61 years of age (31%) (p<0.001).

## Conclusions

Although the infections in the patients in this review were primarily diarrheal, the relative percentage of extraintestinal infections (29%) as well as the ratio (0.42) to diarrheal cases is intriguing. In 2009, only 15% (898/5,888, ratio 0.20) of nontyphoidal subspecies I salmonellae were isolated from extraintestinal sites. Countries other than the United States have reported markedly lower numbers (2%–3%) of nontyphoidal subspecies I extraintestinal infections than subspecies II–IV infections (*2**,**3*). Weiss et al. (*4*), at the US Centers for Disease Control and Prevention (CDC), described a similar high frequency of extraintestinal infections (24%, ratio 0.32) for the Arizona group (now subspecies IIIa and IIIb) between 1967 and 1976. In their review, they ascribed an enhanced virulence for humans to certain serotypes and noted the prevalence of those serotypes within the Arizona group. Within subgroup I, a limited number of certain serotypes are recognized as having a higher propensity for causing extraintestinal disease on the basis of distinct virulence-associated characteristics. These serotypes include Typhi, Dublin, Cholerasuis, and Paratyphi A and C. In contrast, little information is available regarding comparable pathogenicity traits in *Salmonella* subspecies II–IV, with the exception of the Arizona group.

Data available did not enable us to determine how these patients acquired their infections. However, exotic pets are well recognized as a source of infection caused by unusual subspecies of salmonellae (*5**–**7*). Similarly, the most current salmonellae data available from CDC show that 65% of all subspecies II–IV strains were obtained from reptiles (*1*).

The distribution of subspecies II–IV infections by patient sex in this study differs somewhat from that of subspecies I, according to CDC records, which show that subspecies I infections, in general, are slightly more common in female patients (*1*). In our data, overall, male patients were more likely than female patients to be infected with subspecies II–IV salmonellae. This situation was particularly true for subspecies IIIa. Subspecies II and IIIb extraintestinal infections were an exception to this trend; cases were more prevalent in female patients than in male patients, and in both groups, subtypes were predominantly isolated from urine rather than blood.

Most reports in the literature emphasize the prevalence of exotic pet–associated subspecies II–IV infections in children <1 year of age (*8**–**12*). Our data showed an equivalent number of cases in children <1 year of age and in persons 11–60 years of age (from isolates obtained from fecal samples), but the preponderance of extraintestinal infections occurred in the older patient groups. These patients, as owners or handlers of exotic pets, may be exposed to a greater inoculum, whereas children <1 year primarily acquire infections secondarily from fomites or surfaces such as sinks used previously to bathe reptiles or by transmission from handler to child (*5**,**12*).

Little information is available regarding this small, but obviously substantially pathogenic, group of organisms. Our report and those of others (*4*) clearly show that *Salmonella* subspecies II–IV serotypes are capable of causing serious infections, including septicemia and wounds or abscesses. Unfortunately, despite recommendations to the US public, beginning in the mid-1990s, regarding the potential risk for acquisition of salmonellae infections from exotic pets, the number of infections in the United States caused by subspecies associated with these sources does not appear to be abating (*9**,**12*). In California, for subspecies IIIa isolates alone, which are predominantly associated with reptiles, the number of infections doubled from 1993 to 1997 and from 2005 to 2009 (68 vs. 147, respectively). We hope this report will stimulate further epidemiologic investigations into these infections and that this information can then be used to generate a more effective strategy that public health agencies and the exotic pet industry can implement to reduce the extent of disease caused by these organisms.
